# Special Issue “Structure–Activity Relationship of Natural Products”

**DOI:** 10.3390/molecules22050697

**Published:** 2017-04-27

**Authors:** Jean-Marc Sabatier

**Affiliations:** Laboratory INSERM UMR 1097, Aix-Marseille University, 163, Parc Scientifique et Technologique de Luminy, Avenue de Luminy, Bâtiment TPR2, Case 939, 13288 Marseille, France; sabatier.jm1@libertysurf.fr

This Special Issue of *Molecules* deals with the structure–activity relationship of natural compounds which possess some pharmacological/chemical properties of potential interest (from basic research to the clinical applications) in a wide range of areas, such as bacteriology, parasitology, cancerology, inflammation, etc.

The molecules that have been studied in the eleven published articles of the Special Issue are mainly derived from plants and are of a varied nature/complexity, ranging from glycyrrhizic acid to alkaloids. Echeverría, J. et al. [1] focus on the antibacterial properties of natural (plant-derived) flavones and flavanones. The potential of the natural flavonoid phloretin as an antimycobacterial agent (anti-tuberculosis), as well as an anti-inflammatory compound, was also investigated by Jeon and collaborators [2]. The capacity of the natural plant, pterostilbene, and its derivatives to act as anti-biofilm agents against *Candida albicans* was described by Hu et al. [3]. Finally, the antiparasitic (antiprotozoal) activity of triazole derivatives of dehydroabietic and oleanolic acids was reported by Pertino et al. [4]. Activities other than antimicrobial, such as anti-inflammatory activities, were further described by Li et al. [5] for plant diterpenoid alkaloids, and Nam et al. [6] for anthraquinone and its hydroxy derivatives, i.e. purpurin, anthrarufin and chrysazin (the latter also exhibiting antioxidative effects). The antioxidant properties of ferulates was investigated by Karamac et al. [7] whereas the free radical scavenging activity of soy isoflavone glycosides (Daidzin and Genistin)—and their 3’hydroxylated derivatives—was reported by Chiang et al. [8]. Such radical-scavenging activity, together with anti-tumor effects, were further described for *Xanthoceras sorbifolia* bunge/yellow horn plant polyphenols by Yang et al. [9]. Another interesting research article by Singh et al. [10] focused on the potential of glycyrrhizic acid to help both heart contractions and blood pressure. Finally, the last article of this Special Issue, by Liu et al. [11], dealt with the change in molecular content (organic acids, soluble sugars, anthocyanins, etc.) of the Chinese apple cultivar “Starkrimson” during the ripening period.

All these articles are actually highlighting the great potential of natural compounds—and their structural analogs—to act as candidate drugs to treat various diseases, including microbial infections. I would like to thank the authors for their outstanding work in the field of natural compounds.
